# Piperlongumine Is an NLRP3 Inhibitor With Anti-inflammatory Activity

**DOI:** 10.3389/fphar.2021.818326

**Published:** 2022-01-12

**Authors:** Jie Shi, Yang Xia, Huihong Wang, Zhongjie Yi, Ruoruo Zhang, Xiufeng Zhang

**Affiliations:** ^1^ Department of Respiratory Medicine, Second Affiliated Hospital of Hainan Medical University, Haikou, China; ^2^ Department of General Surgery, The Third Xiangya Hospital of Central South University, Changsha, China; ^3^ Department of Plastic and Aesthetic (Burn) Surgery, The Second Xiangya Hospital of Central South University, Changsha, China; ^4^ Institute of Transplantation Medicine, Second Affiliated Hospital of Hainan Medical University, Haikou, China

**Keywords:** piperlongumine, NLRP3, Nek7, inflammasome assembly, inflammation

## Abstract

Piperlongumine (PL) is an alkaloid from *Piper longum* L. with anti-inflammatory and antitumor properties. Numerous studies have focused on its antitumor effect. However, the underlying mechanisms of its anti-inflammation remain elusive. In this study, we have found that PL is a natural inhibitor of Nod-like receptor family pyrin domain-containing protein-3 (NLRP3) inflammasome, an intracellular multi-protein complex that orchestrates host immune responses to infections or sterile inflammations. PL blocks NLRP3 activity by disrupting the assembly of NLRP3 inflammasome including the association between NLRP3 and NEK7 and subsequent NLRP3 oligomerization. Furthermore, PL suppressed lipopolysaccharide-induced endotoxemia and MSU-induced peritonitis *in vivo*, which are NLRP3-dependent inflammation*.* Thus, our study identified PL as an inhibitor of NLRP3 inflammasome and indicated the potential application of PL in NLRP3-relevant diseases.

## Introduction

Piperlongumine (PL) is a natural product from the fruit of long pepper and a form of traditional Chinese medicine (Wang et al., 2014). PL exhibits antitumor properties in serials of tumors including sarcoma, melanoma, gastrointestinal cancers, and bladder cancers by induction of autophagy, apoptosis, and cell cycle arrest through modulating ROS production (Chen et al., 2019; Rawat et al., 2020; Shin et al., 2020). Recent studies have found that PL shows potent anti-inflammatory effects in ovalbumin-induced asthma and airway inflammation, neuroinflammation, and psoriasis-like skin inflammation (Gu et al., 2018; Kim et al., 2018; Lu et al., 2019). However, the underlying mechanisms for PL anti-inflammation were all attributed to the NF-κB signal inhibition. Given the broad anti-inflammatory effects of PL, we speculated that there still exists an unknown mechanism for PL in suppressing inflammatory responses.

The NLRP3 inflammasome is an intracellular multiprotein complex that is critical in protecting the host from infections or sterile injuries (Mao et al., 2013). NLRP3 can sense diverse stimuli including pathogen components, environment irritants, and host danger effectors, so its aberrant activation leads to many inflammatory diseases, such as sepsis (Mao et al., 2013), gout (Martinon et al., 2006), type 2 diabetes (Masters et al., 2010), atherosclerosis (Duewell et al., 2010; Bai et al., 2021), and Alzheimer’s disease (Heneka et al., 2013). It consists of a sensor, a nucleotide-binding domain, a leucine-rich repeat, pyrin domain-containing protein 3 (NLRP3), an adaptor, the apoptosis-associated speck-like protein containing a CARD (ASC), and an effector, caspase-1 (Swanson et al., 2019). NLRP3 inflammasome activation is a two-step process. First, it needs a priming signal to upregulate the expression of NLRP3 and pro-IL-1β, and the priming signal can be induced by various pathogen-associated molecular patterns (PAMPs) or through cytokines such as the tumor necrosis factor (TNF). Second, the inflammasome is formed and fully activated, which can be triggered by a wide variety of stimuli. Oligomerized NLRP3 recruits ASC and then forms a large complex to activate caspase-1, which induces the maturation of IL-1β and IL-18 as well as gasdermin D-mediated pyroptotic cell death.

In this study, we found that PL could inhibit the NLRP3 inflammasome activation in murine and human macrophages. Moreover, PL alleviated the lipopolysaccharide (LPS)-induced endotoxemia and MSU-induced peritonitis *in vivo*, which are NLRP3-dependent inflammations*.* Mechanistically, PL blocks NLRP3 inflammasome assembly by interrupting the interaction between NLRP3 and NEK7 and subsequent aggregation of NLRP3. Thus, our study identified PL as an NLRP3 inhibitor and indicated the potential application of PL in NLRP3-relevant diseases.

## Materials and Methods

### Animals

Wild-type (WT) C57BL/6 mice (8–10 weeks old, weight between 20–25 g) were bought from Hunan SJA Laboratory Animal Co., Ltd. (Changsha, China) and were kept under SPF conditions with standard chows and a 12- h light/dark cycle. All animal experiments were conducted in accordance with Animal Research: Reporting of *In Vivo* Experiments guidelines (Percie du Sert et al., 2020) and the Institutional Animal Care and Use Committee of Central South University.

### Reagents and Antibodies

#### Reagents

Standard LPS (E. coli 0111:B4, Cat No. tlrl-eblps), ultrapure LPS (E. coli 0111:B4, Cat No. tlrl-3pelps), nigericin (Cat No. tlrl-nig), ATP (Cat No. tlrl-atpl), and MSU (Cat No. tlrl-msu) were purchased from InvivoGen (San Diego, CA, United States); the cell lysis buffer (CLB) (Cat No. 9803) was bought from Cell Signaling Technology (Danvers, MA, United States); the mouse immunoglobin IgG protein (Cat No. ab198772) was purchased from Abcam (Cambridge, CB2 0AX, United Kingdom); Protein A/G PLUS-Agarose (Cat No. sc-2003) was obtained from Santa Cruz (Santa Cruz, CA, United States); mouse IL-1β (Cat No. 88–7013), tumor necrosis factor-α (TNF-α) (Cat No. 88-7324), interleukin-6 (IL-6) (Cat No. 88-701364), and a human IL-1β (Cat No. BMS22) ELISA kit was bought from Thermo Fisher (Waltham, MA United States); and the CellTiter-Glo^®^ Luminescent Cell Viability Assay (Cat No. G7572) was from Promega.

### Antibodies

Anti-β-actin (1:10,000, BH10D10) was bought from Cell Signaling Technology (Danvers, MA, United States); Anti-NLRP3 (1:1,000. Cryo-2) and Anti-ASC (1:1,000, AL177) were purchased from Adipogen (San Diego, CA, United States); Anti-Caspase-1 (1:1,000, ab179515) and Anti-NEK7 (1:10,000 ab133514) were bought from Abcam (Cambridge, CB2 0AX, United Kingdom); Anti-IL-1β (1:000 AF-401-NA; RRID: AB_416684) was obtained from RD systems (Tustin, CA, United States); the DyLight 488-labeled secondary antibody (1:50, A120-100D2) was purchased from InvivoGen (San Diego, CA, United States); and FITC anti-mouse/human CD11b (101216, 1:500 for flow cytometry) and APC anti-mouse Ly-6G (127614, 1:500 for flow cytometry) were from BioLegend.

### Cell Culture

THP-1 cells were obtained from American Type Culture Collection (Manassas, VA). C57BL/6 mice were injected intraperitoneally with 3% thioglycolate before collecting primary peritoneal macrophages. Peritoneal lavage was performed to harvest exudate cells and seeded in 48-well (2–3 × 10^5^) or 6-well (2 × 10^6^) culture plates. After 2 h, the non-adherent cells were removed; the adherent monolayer cells were peritoneal macrophages. Primary peritoneal macrophages and THP-1 cells were cultured in the RPMI-1640 medium supplemented with 10% fetal bovine serum, 100 U/ml penicillin, and 100 μg/ml streptomycin at 37 C in a humidified incubator of 5% CO_2_.

### Cell Viability Assay

Peritoneal macrophages and THP-1 cells were seeded in 96-well (4 × 10^4^) culture plates. After treatment with PL (1, 5, 10, 20, and 40) for 30 min, 100 μL of CellTiter-Glo^®^ Reagent was added to each well. We incubated the plate at room temperature for 10 min and recorded luminescence.

### Inflammasome Activation

As previously reported (Wang et al., 2021), for NLRP3 inflammasome activation, macrophages were primed with LPS (100 ng/ml) for 3 h, followed by PL or DMSO for 30min and stimuli as follows: 5 mM ATP or 10 μM nigericin for 1h and 200 μg/ml MSU for 6 h; differentiated adherent THP-1 cells were induced by 100 nM PMA (phorbol-12-myristate-13-acetate) for 3 h and then primed with LPS (1 μg/ml) for 3 h, followed by NLRP3 inflammasome activation stimulation: 5 mM ATP or 10 μM nigericin for 1 h or 200 μg/ml MSU for 6 h.

### ASC Oligomerization

C57BL/6 mice peritoneal macrophages were primed with LPS for 3 h, treated with PL or DMSO for 30min, and stimulated with nigericin for 1h, and then, the cells were lysed with the Triton buffer [50 mM Tris-HCl (pH 7.5), 150 mM NaCl, 0.5% Triton X-100] mixed with 0.1 mM phenylmethylsulfonyl fluoride (PMSF) and the EDTA-free protease inhibitor cocktail for 10 min on ice. Then, the cell lysates were centrifuged at 6000 g for 15 min on ice to collect the supernatant and to resuspend pellets in the 200 μL Triton buffer after washing twice. 2 mM disuccinimidyl suberate (DSS) was added into the resuspended pellets and cross-linked for 30 min at 37°C. All samples were dissolved in the sodium dodecyl sulfate (SDS) loading buffer and heated to 100°C for 10 min for protein denaturation so as to prepare for Western blotting.

### ASC Speck Formation

C57BL/6 mice peritoneal macrophages were seeded on chamber slides overnight. Then, macrophages were primed with LPS for 3 h and treated with PL or DMSO for 30 min and stimulated with nigericin or ATP for 1 h. After that, the cells were fixed in 4% paraformaldehyde (PFA) for 10 min, permeabilized with 0.1% Triton X-100 for 10 min, and blocked with 3% BSA in PBS for 1 h. Cells were then stained with Anti-ASC (1:200 at 4°C overnight) and the DyLight 488-labeled secondary antibody (1:50 at room temperature for 45 min). Macrophage nuclei were dyed with DAPI. A fluorescence microscope (Nikon Ti2-U) was used to check these stained cells and ASC specks.

### Immunoprecipitation and Western Blot

After indicated nigericin stimulation for 1 h, mice peritoneal macrophages were lysed in an immunoprecipitation (IP) buffer mixed with PMSF and the cocktail. Then, these cell lysates were reacted to specific antibodies ASC or NEK7 and protein G plus-agarose overnight and washed four times with the IP buffer. Immunoprecipitates were eluted by boiling with 1% (w/v) SDS loading buffer.

The supernatants (SN) were immunoprecipitated with NLRP3 antibodies for 12 h at 4°C and protein A/G agarose for 2 h. The immunoprecipitants were washed six times with the IP buffer and boiled with 1% (w/v) SDS loading buffer for 10 min for immunoblot analysis.

For Western blot, stimulated macrophages were lysed with CLB (CST) supplemented with the cocktail and PMSF and subsequently centrifuged at 12,000 g at 4°C for 10 min. Protein concentrations were detected with a bicinchoninic acid assay (Pierce). An equal content of extracts was separated by SDS-PAGE and transferred onto 0.22-mm PVDF membranes (Merck Millipore).

### SDD-AGE

Western blot of the NLRP3 aggregate was analyzed following published protocols (Hou et al., 2011; Jiang et al., 2017). The procedure is briefly described as follows: mice peritoneal macrophages were lysed with the Triton X-100 lysis buffer, supplemented with PMSF and the cocktail, and then centrifuged at 12,000 g at 4°C for 5 min. Next, the cell lysates were resuspended in a 5 × sample buffer (2.5 × TBE, 50% glycerol, 10% SDS, and 0.0025% bromophenol blue) and run onto vertical 1.5% agarose gel. After electrophoresis for 1 h at a constant voltage of 80 V at 4°C in the running buffer (1 × TBE and 0.1% SDS), the proteins were transferred onto 0.22-mm PVDF membranes for 1 h for the following immunoblot.

### ELISA Assay for Cytokines

Levels of IL-1β, IL-6, and TNF-α obtained from cell culture after stimulations and mice blood serum were detected in quantitative ELISA kits (eBioscience), according to the manufacturer’s instructions.

### LDH Release Assay

Levels of LDH release in cells after stimulations were determined using an LDH Cytotoxicity Assay Kit bought from Beyotime (Shanghai, China), according to the manufacturer’s instructions.

### *In vivo* Endotoxemia Model

Wild-type C57BL/6 mice were pretreated with PL (50 mg/kg or 100 mg/kg) or an empty solvent (as an empty control) for 0.5 h and then injected intraperitoneally with LPS (20 mg/kg). After 8 h, mice were sacrificed; the blood serum was collected by heart puncture to detect concentrations of IL-1β, IL-6, and TNF-α by ELISA; and the lungs were harvested for histology analysis.

### MSU-Induced Peritonitis *In Vivo*

Wild-type C57BL/6 mice were pretreated with PL (100 mg/kg) or an empty solvent (as an empty control) for 0.5 h. Next, they were injected intraperitoneally with 1 mg MSU (dissolved in 500 μL PBS) for 6 h. Peritoneal lavage was performed using 10 ml ice-cold PBS to collect peritoneal exudate fluids and concentrated for ELISA analysis with an Amicon Ultra 10 K filter (UFC900308) from Millipore. Peritoneal cells were collected and analyzed by flow cytometry.

### Lung W/D Weight Ratio

The severity of pulmonary edema was estimated by calculating the lung wet/dry (W/D) weight ratio. After sacrifice, the left lobe of the lung was excised, washed with phosphate-buffered saline (PBS), and weighed to gain the “wet” weight. The left lung was then placed in an oven for approximately 72 h at 65°C until there were no changes in the weight to obtain the “dry” weight.

### Histological Analysis

After PBS perfusion to the cardiac, the lower right lobe of the lung was cut and fixed in 4% paraformaldehyde solution at room temperature for 24 h. After regular dehydration for histological sections, these specimens were embedded with paraffin. Next, sections were cut and mounted on polysine adhesion glass slides for subsequent hematoxylin and eosin staining using standard procedures. Slides were examined under a Nikon ECL IPSE Ci biological microscope, and images were captured with a Nikon DS-U3 color digital camera.

### Statistical Analysis

All values in our experiments are shown as the mean ± SD. Statistical analysis was performed using GraphPad Prism 8.0 software. Unpaired Student’s t test was used for comparison of two groups. When comparing more than two groups, ANOVA with the Bonferroni test was used. The statistical significance was set at *p* < 0.05.

## Results

### Piperlongumine Inhibits NLRP3 Inflammasome Activation in Mouse Macrophages

**FIGURE 1:**
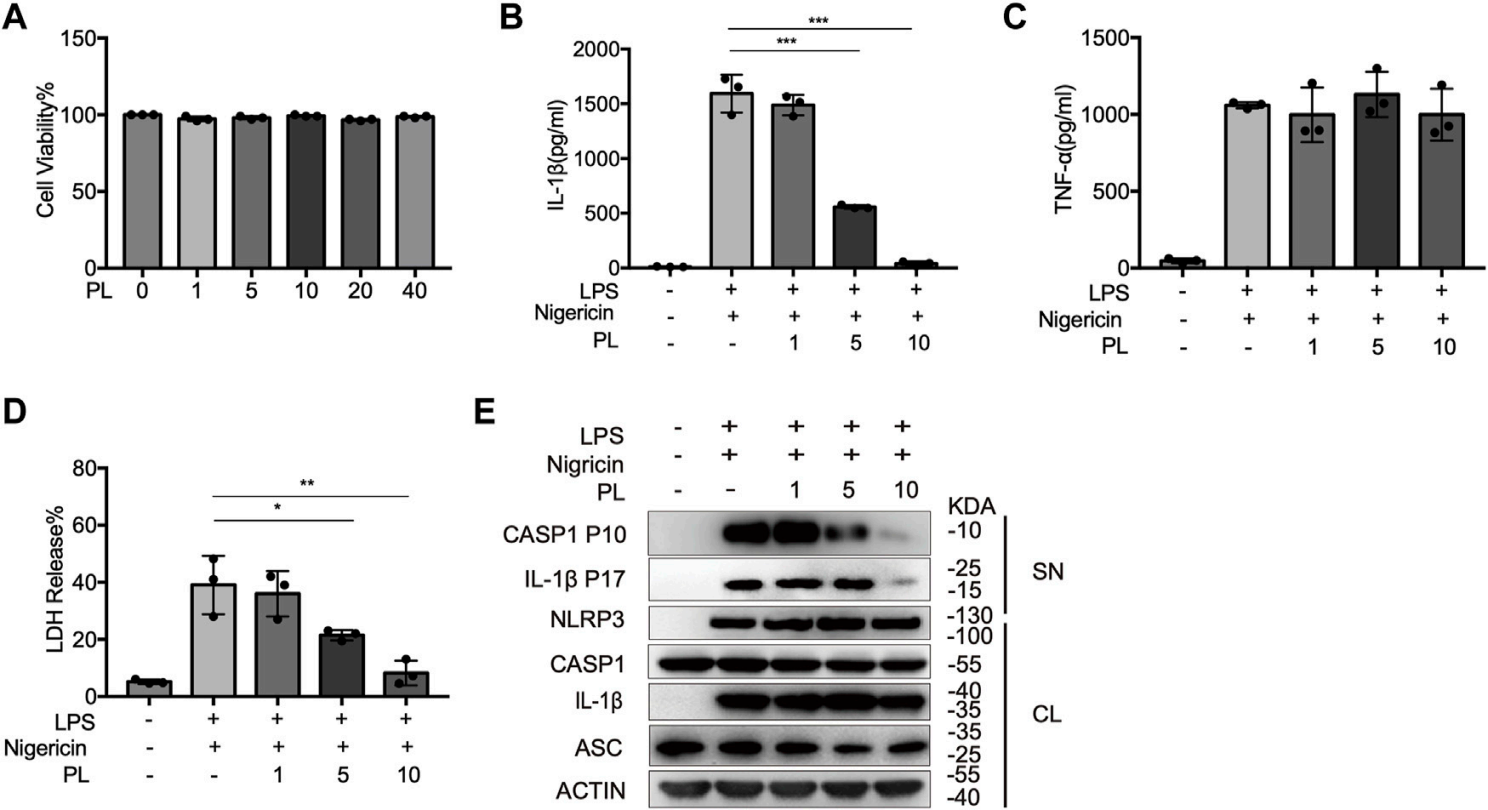
PL dose-dependently inhibits nigericin-induced NLRP3 inflammasome activation. **(A)** Cell viability of PL (1–40 μM) in peritoneal macrophages. **(B–D)** ELISA of IL-1β **(B)**, TNF-α **(C)**, and release of LDH **(D)** in supernatants from LPS-primed mouse peritoneal macrophages treated with 1–10 μM PL and stimulated with nigericin. **(E)** Immunoblot of supernatants or cell lysates from LPS-primed mouse peritoneal macrophages treated with 1–10 μM PL and stimulated with nigericin. All data were representative of three independent experiments. Values shown are mean ± SD. For statistical analysis, A–D were analyzed using one-way ANOVA and the Bonferroni test. *p < 0.05; **p < 0.01; ***p < 0.001.

We first examined the cytotoxicity of PL (1–40 μM) by cell viability and proved that the doses of PL were not cytotoxic (Figure 1A). To explore whether PL inhibits NLRP3 inflammasome, we treated LPS-primed mouse peritoneal macrophages with PL to exclude the effects of PL on the priming signal and then added nigericin, an NLRP3 agonist by causing K+ efflux. Interestingly, PL exhibited dose-dependent inhibitory effects on LPS + nigericin-induced IL-1β secretion and LDH release at the doses of 1–10 μM, while it had no effect on inflammasome-independent cytokine TNF-α production (Figures 1B–D). Similarly, the cleaved caspase-1 (p10) was reduced dose-dependently, measured by Western blot. Moreover, PL barely affected the expression of NLRP3, ASC, the precursors of IL-1β, or the precursors of caspase-1 (Figure 1E).

**FIGURE 2:**
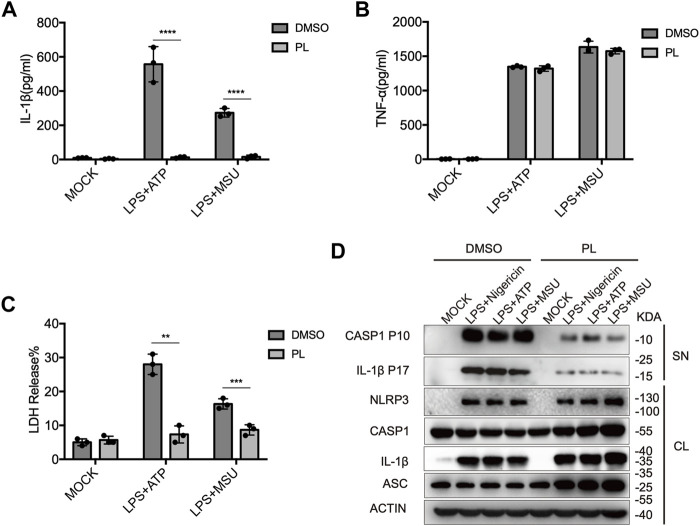
PL inhibits ATP or MSU-induced NLRP3 inflammasome activation. **(A–C)** ELISA of IL-1β **(A)**, TNF-α **(B)**, and release of LDH **(C)** in supernatants from LPS-primed mouse peritoneal macrophages treated with 10 μM PL or DMSO and stimulated with ATP or MSU. **(D)** Immunoblot of supernatants or cell lysates from LPS-primed mouse peritoneal macrophages treated with 10 μM PL and stimulated with indicated stimuli. All data were representative of three independent experiments. Values shown are mean ± SD. For statistical analysis, A, B, and C were analyzed using two-way ANOVA and the Bonferroni test; **p < 0.01; ***p < 0.001; ****p < 0.0001.

We further observed that PL inhibited IL-1β secretion, LDH release, and caspase-1 cleavage when macrophages were treated with other NLRP3 agonists, including ATP and MSU (Figures 2A–D). Taken together, these results demonstrated the inhibitory effects of PL on the NLRP3 inflammasome in mouse macrophages.

### Piperlongumine Suppresses NLRP3 Inflammasome Activation in THP-1 Cells

**FIGURE 3:**
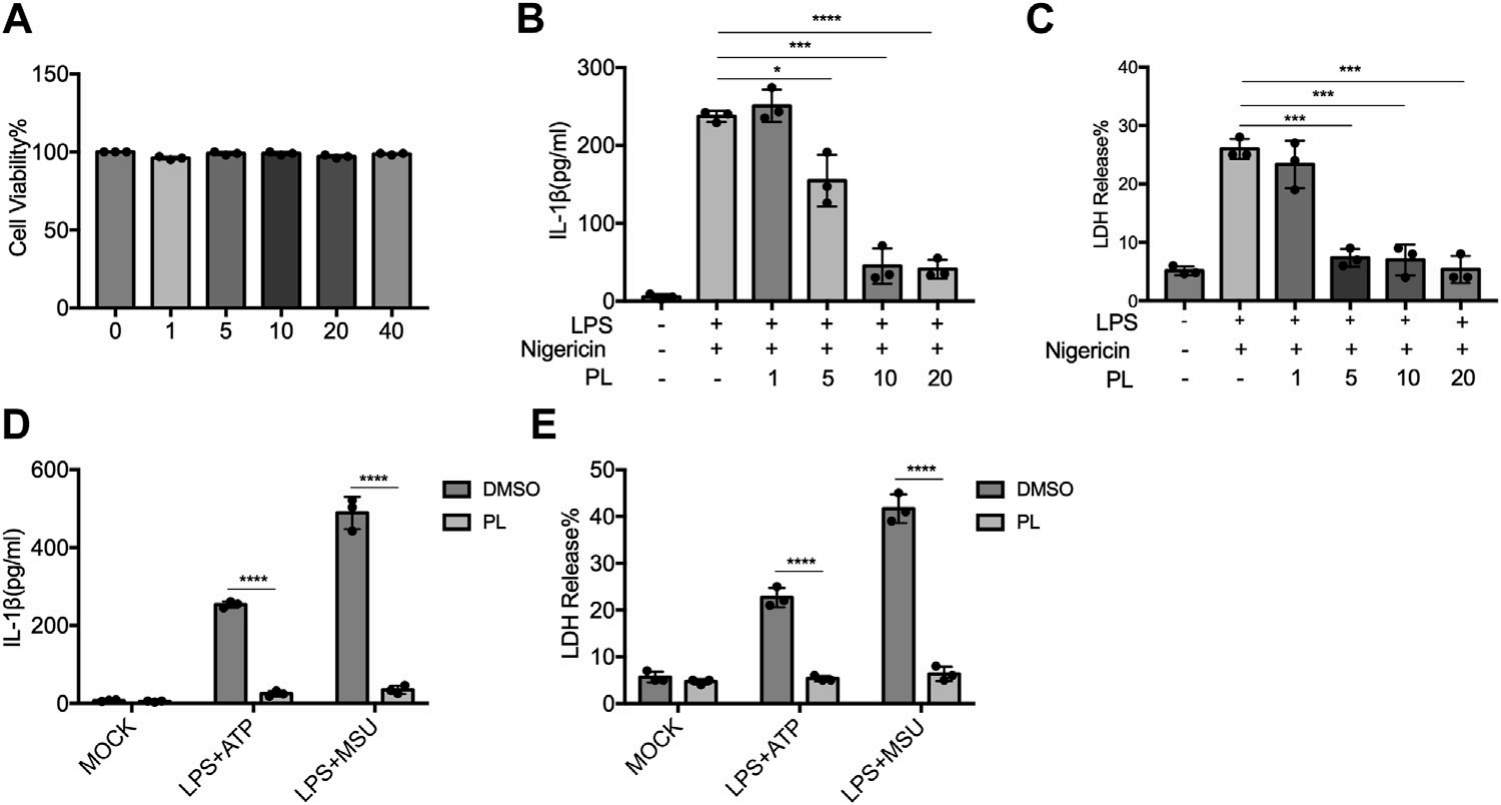
PL blocks NLRP3 inflammasome activation in THP-1 cells. **(A)** Cell viability of PL (1–40 μM) in THP-1 cells. **(B–C)** ELISA of IL-1β **(B)** and release of LDH **(C)** in supernatants from PMA-primed THP-1 cells treated with 1–10 μM PL and challenged with nigericin. **(D–E)** ELISA of IL-1β **(D)** and release of LDH **(E)** in supernatants from PMA-primed THP-1 cells treated with 10 μM PL or DMSO and stimulated with ATP or MSU. Values shown are mean ± SD. For statistical analysis, A–C were analyzed using one-way ANOVA and the Bonferroni test. D and E were analyzed using two-way ANOVA and the Bonferroni test; *p < 0.05; ***p < 0.001; ****p < 0.0001.

To further examine whether PL inhibits NLRP3 inflammasome in human cells, we detected the effects in THP-1 cells. First, we detected the cytotoxicity of PL (1–40 μM) by cell viability and proved that the doses of PL were not cytotoxic (Figure 3A). Treating PL with PMA-primed THP-1 cells, we observed the declined IL-1β secretion and LDH release when challenged with nigericin, ATP, and MSU (Figures 3B–E). Thus, PL exerts an inhibitory role in NLRP3 inflammasome activation in human cells.

### Piperlongumine Interrupts ASC Speck Formation

**FIGURE 4:**
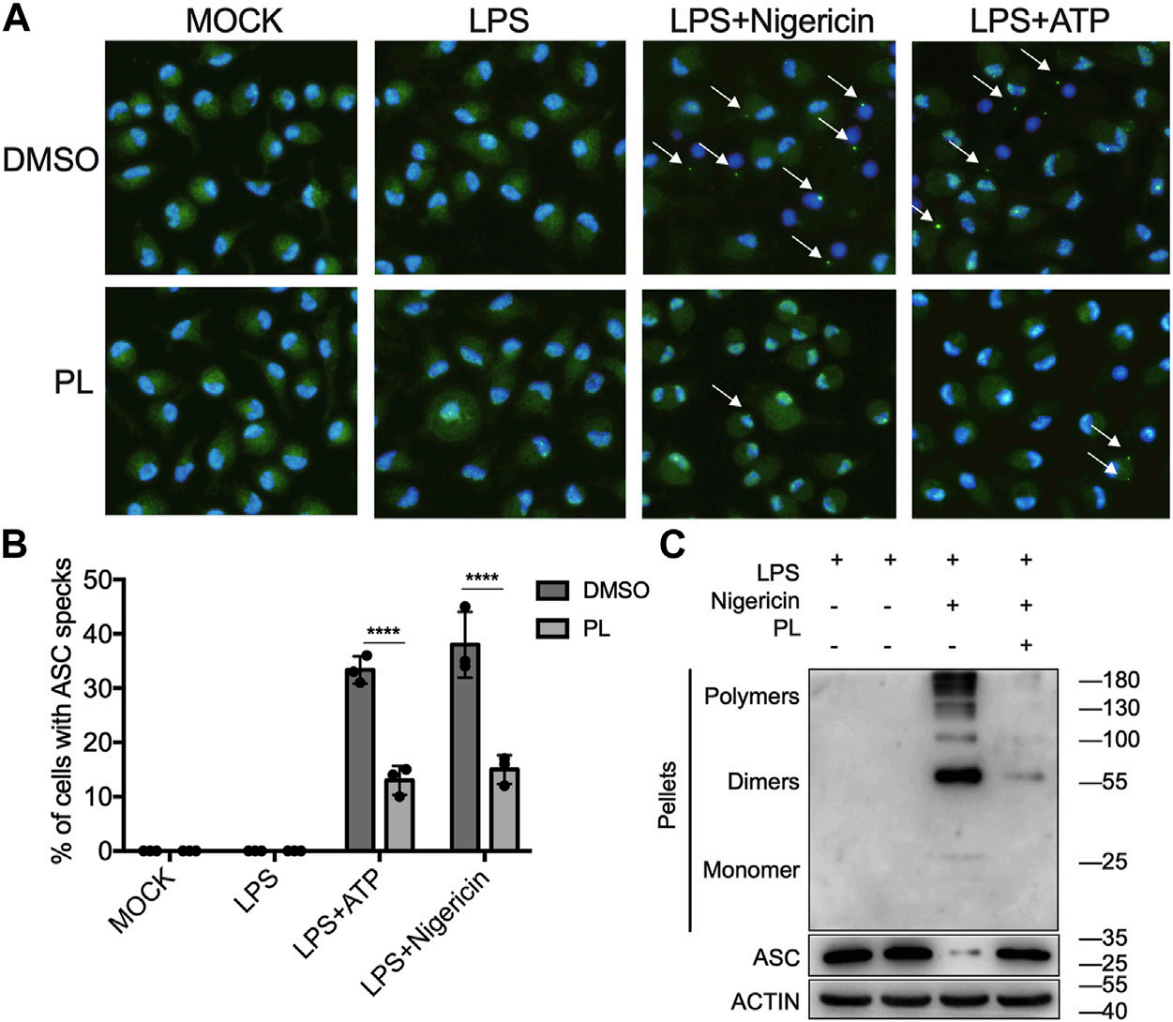
PL suppresses ASC speck formation. **(A, B)** Immunofluorescence microscopy analysis of ASC specks in LPS-primed mouse peritoneal macrophages treated with 10 μM PL or DMSO and stimulated with ATP or nigericin. **(A)** Representative images of ASC speck distribution in cells; ASC, green; nuclei, blue. White arrows indicate ASC specks. **(B)** Quantified percentage of cells containing an ASC speck. At least 100 peritoneal macrophages were collected for analysis. **(C)** Immunoblot analysis of ASC oligomerization in cross-linked cytosolic pellets of LPS-primed mouse peritoneal macrophages treated with 10 μM PL and then stimulated with nigericin. Values shown are mean ± SD. For statistical analysis, two-way ANOVA and the Bonferroni test were used; ****p < 0.0001. Data were collected from three independent experiments.

Next, we explored how PL inhibits NLRP3 activation. ASC speck formation is an essential step for NLRP3 activation (Oroz et al., 2016; Green et al., 2018), and then, we intended to determine whether PL has a regulatory role in ASC speck formation. With immunofluorescence microscopy analysis, we observed that PL markedly decreased the percentage of macrophages containing the ASC speck after stimulated with nigericin or ATP (Figures 4A,B). In common with the results of microscopy, PL distinctly reduced appearance of large multimeric ASC complexes in chemical cross-linking agents by Western blot (Figure 4C). Thus, the results indicated that PL blocks ASC oligomerization.

### Piperlongumine Inhibits NLRP3 Inflammasome Assembly

**FIGURE 5:**
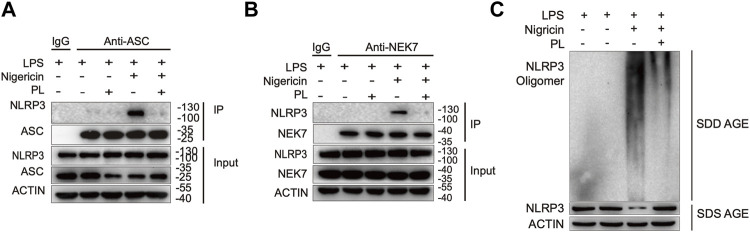
PL interrupts NLRP3 inflammasome assembly. **(A)** Immunoblot analysis (immunoprecipitation) of the interaction between NLRP3 and ASC in LPS-primed mouse peritoneal macrophages treated with 10 μM PL or DMSO and then stimulated with nigericin. **(B)** Immunoblot analysis (Immunoprecipitation) of the interaction between NEK7 and NLRP3 in LPS-primed primary macrophages treated with 10 μM PL or DMSO and then stimulated with nigericin. **(C)** Immunoblot analysis of NLRP3 oligomerization using SDD-AGE or SDS-PAGE assays in LPS-primed mouse peritoneal macrophages treated with 10 μM PL or DMSO and then stimulated with nigericin.

Since ASC speck formation is a result of ASC recruitment to NLRP3 (Martinon et al., 2009; Davis et al., 2011), we next investigated whether PL influenced the interaction between them. By performing immunoprecipitation of ASC and NLRP3, we observed that PL markedly interrupted the ASC-NLRP3 association (Figure 5A), suggesting that PL targets the upstream of recruitment of ASC to NLRP3. Before recruiting ASC, NLRP3 first aggregates with the help of NEK7, a newly described component of NLRP3 inflammasome (He et al., 2016). We then detected the interaction between NEK7 and NLRP3. When treated with PL, the NEK7-NLRP3 association was disrupted (Figure 5B). Accordingly, the endogenous oligomerization of NLRP3 was dramatically decreased by using semi-denaturing detergent agarose gel electrophoresis (SDD-AGE) (Figure 5C). Thus, PL suppresses NLRP3 inflammasome activation through inhibiting the NLRP3 inflammasome assembly.

### Piperlongumine Suppresses NLRP3-Dependent Inflammation *in vivo*

**FIGURE 6:**
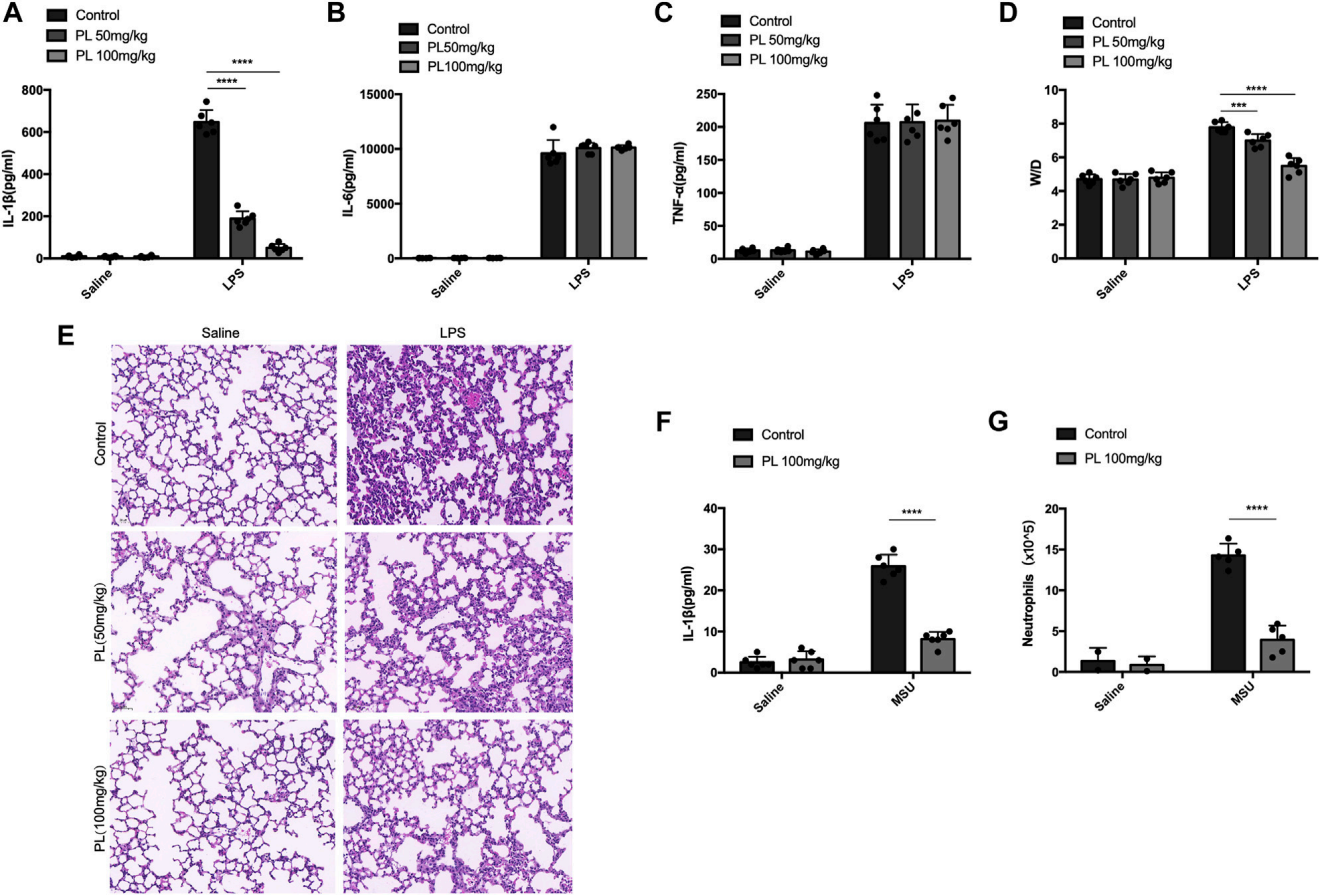
PL disrupts NLRP3 inflammasome activation *in vivo*. **(A–E)** Effects of PL in endotoxemia. Wild-type mice with the C57BL/6 background were pretreated with intraperitoneal injection of PL (50 mg/kg or 100 mg/kg) or N.S 30 min before intraperitoneal injection of LPS (20 mg/kg) for 8 h. ELISA analysis of IL-1β **(A)**, IL-6 **(B)**, and TNF-α **(C)** was performed in mice blood serum. **(D)** Lung W/D ratio of endotoxemic mice treated with PL (50 mg/kg or 100 mg/kg). **(E)** Representative images of HE staining in lungs. **(F, G)** Effects of PL in MSU-induced peritonitis. Wild-type mice with the C57BL/6 background were intraperitoneally injected of PL (100 mg/kg) or N.S 30 min before intraperitoneal injection of MSU (1 mg) for 6 h. ELISA of IL-1β **(F)** and neutrophils (flow cytometry) **(G)** in the peritoneal cavity fluid were quantified. Values presented are mean ± SD. Two-way ANOVA and the Bonferroni test were used for the statistical analysis; ***p < 0.001; ****p < 0.0001. Data were collected from three independent experiments.

Finally, we investigated whether PL could inhibit NLRP3 inflammasome activation *in vivo*. Intraperitoneal injection of LPS or MSU induces IL-1β secretion and neutrophil infiltration in a NLRP3-dependent manner (Martinon et al., 2006). Pretreatment of PL (50 mg/kg or 100 mg/kg) could markedly attenuate release of IL-1β without affecting IL-6 and TNF-α in serum induced by LPS injection (Figures 6A–C). Moreover, the PL-treated group showed moderate lung edema by calculating the W/D ratio (Figure 6D) and smaller bleeding spots, less inflammatory cell infiltration, and less impaired structures in lungs evaluated by histopathology, compared to the control group (Figure 6E). In another MSU-induced peritonitis model, PL also exhibited inhibitory effects on NLRP3 inflammasome reflected by reduced IL-1β (Figure 6F) and recruitment of neutrophils (Figure 6G) in the lavage fluid. Taken together, these data proved that PL could inhibit NLRP3-dependent inflammation *in vivo*.

## Discussion

Piperlongumine, a kind of amid alkaloids, is an extract from the fruits of long pepper plants in Southern India and Southeast Asia. It not only flavors food tastes but also protects human health. Numerous studies have reported its anticancer function in different types of tumors both *in vitro* and *in vivo*, including colon, pancreatic, gastric, cholangio, lung, and prostate cancers (Randhawa et al., 2013; Dhillon et al., 2014; Ginzburg et al., 2014; Duan et al., 2016; Thongsom et al., 2017; Hałas-Wiśniewska et al., 2020). The anticancer properties of PL were demonstrated through cell cycle arrest, pro-apoptosis, anti-invasiveness, and antiangiogenesis by targeting JAK-STAT, NF-kB, or PI3K/AKT/mTOR pathways (Farooqi et al., 2018; Piska et al., 2018). Recently, a few studies have uncovered the role of PL in alleviating sorts of inflammatory disorders, such as colitis, amyloidogenesis, liver fibrosis, diabetes, and psoriasis-like skin inflammation, suggesting an anti-inflammatory effect of PL (Gu et al., 2018; Chilvery et al., 2020; Thatikonda et al., 2020; Xu P. et al., 2021). In addition, these studies have proved that PL inhibits pro-inflammatory cytokine (TNF-α and IL-6) production mainly through suppressing the NF-κB signal and iNOS expression. However, only this mechanism could not explain the role of PL under so many inflammatory conditions.

**FIGURE 7:**
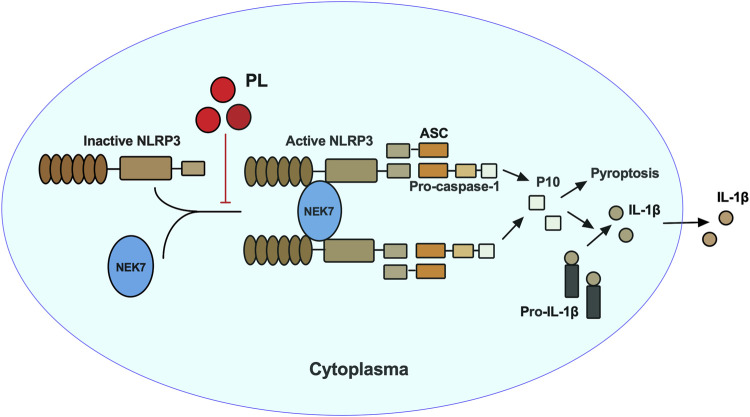
Mechanism of PL inhibits NLRP3 inflammasome activation. PL blocks NLRP3 inflammasome activation by disrupting the interaction between NEK7 and NLRP3 and subsequent NLRP3 oligomerization.

In this study, we demonstrated that PL is an inhibitor of NLRP3 inflammasome (Figure 7). Treated human or murine LPS–primed macrophages with PL could inhibit NLRP3 inflammasome-induced IL-1β production and pyroptotic cell death without affecting inflammasome-independent cytokine TNF-α production. We noted that different from the previous study, PL did not suppress TNF-α production in this model because of the fact that PL addition was after the NF-κB signal activation. Moreover, it presented the specificity of PL in NLRP3 inflammasome without affecting the NF-κB signal. To further demonstrate whether PL could inhibit NLRP3 inflammasome *in vivo*, we first adopted an LPS-induced endotoxemia murine model and observed that PL markedly alleviated the inflammation, which is in line with a previous study (Lee et al., 2013). In another MSU-induced peritonitis model, PL exhibited similar effects by suppressing IL-1β production and neutrophil infiltration, both of which were dependent on NLRP3 inflammasome. Mechanistically, PL could inhibit the NLRP3 inflammasome assembly. By checking the ASC speck, an NLRP3 inflammation activation marker, we noticed that PL may target the upstream of ASC speck formation. Although performing SDD-AGE and immunoprecipitation, we demonstrated that PL interrupted NLRP3 oligomerization and the interaction between NLRP3 and NEK7, a newly recognized partner that bridges the bond of adjacent NLRP3 to form NLRP3 aggregates (He et al., 2016). However, the detailed molecular mechanism for PL that blocks the interaction between NLRP3 and NEK7 is not clear, which still remains further investigation.

A previous study treated macrophages with PL before LPS priming and found that PL could inhibit NLRP3 inflammasome activation through disruption of the NF-κB signaling pathway (Huang et al., 2021). However, in our study, we treated PL after LPS priming to exclude affecting the NF-κB signal, and we found that PL could inhibit NLRP3 inflammasome activation through inhibiting the interaction of NLRP3 and NEK7 rather than the expression of NLRP3. Our study found a different anti-inflammatory mechanism of PL. Taken together, our study and previous study indicated that PL not only widely suppresses inflammatory response through the NF-κB signaling pathway but also specifically inhibits NLRP3 inflammasome activation.

NLRP3 inflammasome is the most well-studied inflammasome. Numerous studies have indicated that excessive NLRP3 inflammasome activation is harmful to the host immune system and can lead to many diseases that are related to the long-term inflammatory process including type 2 diabetes mellitus, atherosclerosis, rheumatoid arthritis, and gout (Swanson et al., 2019). Disruption of NLRP3 inflammasome activation exhibits a therapeutic role to these diseases, thus apparently indicating its promising property in dealing with inflammatory related disorders. Accordingly, several compounds have been discovered for inhibiting NLRP3 inflammasome, and among them, MCC950 is the most well-studied NLRP3 inhibitor. By directly interacting with NLRP3, MCC950 leads to an inactive NLRP3 conformation (Tapia-Abellán et al., 2019). Besides MCC950, there are a serial of inhibitors directly interacting with NLRP3 and inhibiting NLRP3 ATPase activity, including CY-09, Bay 11–7082, OLT1177 dapansutrile, INF39, MNS, and BOT-4-one (Swanson et al., 2019). Apart from directly interacting with NLRP3, there are some inhibitors that control the NLRP3 activation in a posttranslational modification manner, such as SP600125, which disrupts the phosphorylation of NLRP3 and ASC; G5 prohibits the deubiquitination of NLRP3 (Jiang et al., 2020). In addition, some inhibitors target the association between NLRP3 and ASC, such as cardamonin (Jiang et al., 2020), SI-2 (Liu et al., 2020), and C646 (Xu X. et al., 2021), and some inhibitors block the interaction between NLRP3 and NEK7, including ordonin (Swanson et al., 2019) and ginsenoside Rg3 (Jiang et al., 2020). Our study added another natural inhibitor for NLRP3 by interrupting the interaction between NLRP3 and NEK7.

In summary, our study identified PL as an NLRP3 inhibitor by interrupting the assembly of the inflammasome, providing a new view of the anti-inflammatory mechanism of PL. Moreover, given aberrant activation of NLRP3 inflammasome leads to many inflammatory diseases; our study indicated the potential application of PL in NLRP3-related diseases.

## Data Availability

The original contributions presented in the study are included in the article/Supplementary Material; further inquiries can be directed to the corresponding authors.
